# The Intensity of Primary Care for Heart Failure Patients: A Determinant of Readmissions? The CarPaths Study: A French Region-Wide Analysis

**DOI:** 10.1371/journal.pone.0163268

**Published:** 2016-10-11

**Authors:** Claire M. Duflos, Kamila Solecki, Laurence Papinaud, Vera Georgescu, François Roubille, Gregoire Mercier

**Affiliations:** 1 Economic evaluation unit at Montpellier teaching hospital, Montpellier, France; 2 PhyMedExp, University of Montpellier, INSERM U1046, CNRS UMR 9214, 34295 Montpellier cedex 5, France; 3 Department of Cardiology, Montpellier teaching hospital, Montpellier, France; 4 Information Systems Unit at the Regional medical office of Assurance Maladie, Montpellier, France; 5 MACVIA-LR: Fighting Chronic Diseases for Active and Healthy Ageing (Reference Site of the European Innovation Partnership on Active and Healthy Ageing), Montpellier, France; National Cancer Center, JAPAN

## Abstract

**Background:**

We aimed to classify patients with heart failure (HF) by the style of primary care they receive.

**Methods and Results:**

We used the claim data (SNIIRAM: Système National d’Information Inter-Régime de l’Assurance Maladie) of patients living in a French region. We evaluated three concepts. First, baseline clinical status with age and Charlson index. Second, primary care practice style with mean delay between consultations, quantity of nursing care, and variability of diuretic dose. Third, clinical outcomes with death during follow-up, readmission for HF, and rate of unforeseen consultations. The baseline clinical status and the clinical outcomes were included to give an insight in the reasons for, and performance of, primary care practice style. Patients were classified using a hierarchical ascending classification based on principal components. A total of 2,751 patients were included in this study and were followed for a median of 22 months. The mean age was 78 y (SD: 12); 484 (18%) died, and 818 (30%) were readmitted for HF. We found three different significant groups characterized by their need for care and the intensity of practice style: group 1 (N = 734) was “low need-low intensity”; group 2 (N = 1,060) was “high need-low intensity”; and group 3 (N = 957) was “high need-high intensity”. Their readmission rates were 17%, 41% and 28%, respectively.

**Conclusions:**

This study evaluated the link between primary care, clinical status and main clinical outcomes in HF patients. In higher need patients, a low-intensity practice style was associated with poorer clinical outcomes.

## Introduction

Heart failure (HF) affects 1–2% of the general population and 10% of Western inhabitants older than 75 y [1], and it is associated with increased levels of morbidity and mortality, decreased quality of life and increased costs [2]. HF patients are particularly vulnerable to readmission; all-cause readmission rates have been reported as between 5.6% after 30 days and 45% after one year [3,4].

The current proposed theoretical framework for determinants of readmission includes health policy, patient-level factors (age, ethnicity, health status and socioeconomic resources [5]), in- and outpatient access to and quality of care, and interfaces between actors [6,7], which included organizational factors such as transitional care interventions and continuity of care [8]. The extent of the impact of these determinants is related to pathology [9]; HF is an ambulatory care–sensitive condition [10]. Avoiding these admissions by improving the access to and effectiveness of primary care could result in a substantial decrease in costs and could enhance patient outcomes [11].

More precisely, the access to outpatient care has been measured by the density of primary care physicians and nurses and by the number and frequency of general practitioner (GP) visits [12]. Among studies assessing the role of primary care features, ecological analyses are prone to bias [13], and patient-level studies have yielded inconsistent results, possibly because they do not take into account the content of primary care. Indeed, this content depends on practice style [14] and should be adapted to the patient’s health status. In France, where HF is the leading cause of potentially avoidable hospitalizations, the primary care system allows high variations of practice styles, even in local settings. Primary care is mainly delivered by self-employed physicians in the ambulatory care sector. A semi-gatekeeping role, played by GPs, is driven by financial incentives but is not required by law. Hence, the weak coordination between GPs, specialists and hospitalists is regarded as a major weakness of the system. Some programs aiming to improve community care for patients with HF took place in recent years but failed to perpetuate and spread, such as the ICALOR network (Insuffisance CArdiaque en LORraine) [15], or were not properly assessed for efficiency, such as the nation-wide Prado scheme [16].

Nevertheless, the interest in this subject has not decreased, and clinical trials are currently ongoing to address the interest of new devices and organizations for the follow-up of patients with HF [17,18]. An accurate analysis of the efficacy of different practice styles could help to determine the link between primary care and potentially avoidable hospitalizations for HF. This might be used to modify guidelines, to improve medical training, to organize care pathways, and to monitor the impact of these interventions. To date, evidence regarding the impact of primary care practice style is scarce, especially in the French context.

The aim of this study was to evaluate the possibility of classifying patients with HF based on three concepts: baseline clinical status, practice style, and clinical outcomes. Importantly, this classification was built without any predefined conditions or associations to obtain a statistically relevant classification, which we assume to be complementary to the usual clinically relevant classifications.

## Methods

### Data Sources

We used the French national mandatory health insurance database (SNIIRAM), which contains in- and outpatient claim data for all patients and all payers.

### Population

We included all adult patients of the Languedoc-Roussillon region (LR region, 2.7 million inhabitants) who had a first hospitalization for HF in 2012 (index hospitalization). Heart failure was defined using the International Classification of Diseases, 10th edition (ICD-10), diagnostic codes for hospital discharge data [19]. We excluded patients who had a previous hospitalization for HF in the two years preceding the index one (2010–2011), who died during the index hospitalization, or who had less than one year of follow-up. This last exclusion criterion has two advantages. First, it prevents any bias induced by patients dying during the early post-hospitalization phase, when the follow-up by the GP has a lesser impact. Second, it allows us to compute reliable practice style variables, and to include the null values in our analyzes.

### Variables

Patient variables were age, sex, Charlson index [20] (number of comorbidities), recipient of “Couverture Maladie Universelle complémentaire” or “Aide Medicale d’Etat” (CMUc or AME; government health insurance programs for individuals with limited financial resources), and deprivation index at the ZIP code level (“commune” in French) [21]. This index follows a normal distribution, and high values denotes deprived areas. It incorporates the percent of blue collar workers, the percent of graduates of high school, the percent of unemployed people, and the median household income. It is routinely computed by the French National Institute of Statitics and Economic Studies.

We described practice style with visit patterns and medication use patterns. Visit patterns included the following: the delay between discharge from the index hospitalization and the first scheduled GP visit (delay to first GP visit); the mean delay between two GP visits (GP mean delay); the delay between the discharge of the index hospitalization and the first scheduled cardiologist visit (delay to first cardiologist visit); the mean delay between two cardiologist visits (cardiologist mean delay); and the percentage of days with at least one nurse home visit (nursing care index). Medication use patterns included two variables: first, the coefficient of variation of daily loop diuretic intake (diuretic variability), as it could reflect treatment adaptation, performed both by the HF specialist or the GP; and second, the delay to the discontinuation of a therapeutic class of long-term HF treatment. Three classes of long-term treatment were considered as follows: angiotensin-converting enzyme inhibitors or angiotensin receptor blockers (ACE-I/ARB), beta-blockers (BB), and mineralocorticoids receptor antagonists (MRA). These 3 classes of drugs were chosen because they all reduce mortality when appropriately administered; in 2012, ACE-I/ARB and BB were recommended in the latest guidelines, whereas there was sufficient scientific evidence to recommend MRA in NYHA 3 patients, as this was included in the guidelines of 2012. A patient was considered to need one of these classes if it was dispensed within 42 days after the index hospitalization. After this first dispensation, we tracked all subsequent dispensations of any medication in this class; if the n-th dispensation occurred more than 42 days after the (n-1)-th dispensation, the (n-1)-th dispensation was defined as the date of discontinuation of this class. This threshold was graphically chosen on the histogram of inter-dispensations delays as the breaking point of the slope between frequent and rare delays. The delay to treatment discontinuation was finally computed after the earlier date among the dates of discontinuation for ACE-I/ARB’s, BB’s, and MRA’s.

Clinical outcomes included the number of readmissions for HF (HF readmissions), all-cause deaths, and percentage of unforeseen medical contacts (unforeseenness index). The denominator of this index was all hospitalizations and medical visits, and the numerator was emergent hospitalizations, emergency room visits, and emergent or out-of-schedule fee-for-service medical visits. The latter were defined by extra fees for visits between 8 P.M. and 8 A.M., between noon on Saturday and 8 A.M. on Monday, and on public holidays for which physicians could bill patients.

### Statistical analysis

We designated groups of patients using a hierarchical ascendant classification (HAC) based on principal components analysis (PCA) [22]. This cluster analysis discerns patterns and creates groups that have similar characteristics across clustering variables, which are the quantitative variables that had less than 10% missing data. Such methods are widely used in varied sciences (economics, climatology, genomics …) and have already been successfully used to analyze claim data [23] and to classify physician practice styles [14].

The PCA is the basis of multivariate descriptive analysis methods developed in the 1970’s. It allows describing a population for which we have partially correlated variables, without needing to choose *a priori* between these variables. It converts the set of original variables, which are partially correlated, into a set of variables called principal components, or axes, which are linear combinations of the original variables. The components have two characteristics: first, all components are independent from each other; second, the first component has the highest variance, followed by the second, and so forth, until the whole variance of the population is represented. The relations of axes and original variables are displayed numerically by a correlation matrix and graphically by correlation circles; whenever clinically relevant, one can therefore attribute a clinical meaning to an axis from the meaning of the original variables that strongly correlate with it. The most interesting means to display the percentages of variance of each axis is a scree plot. Pragmatically, by interpreting these axes, one can tell (a) which variables are the most important for describing the population (b) which the original variables are strongly correlated with each other and (c) in which the original variables are mostly independent of each other.

These axes also have two advantages, which allow them to be used instead of the original variables to sort the population into clusters. First, because an axis concentrates the variance of the variables that it represents, it has high explanation power. Second, by “summarizing” a group of variables, it is less prone to basal noise and therefore is more stable. Moreover, the last axes, which account for a small amount of variance, can themselves be considered as basal noise. Therefore, as classically performed, we applied our clustering method on the first axes of the PCA. The number of clustering axes was chosen according to the 70% criterion, which recommends keeping enough axes to attain 70% of the total variance.

We chose an HAC, because it is a classical clustering method that does not require the determination of a number of clusters *a priori*. This method starts with clusters defined as the observations themselves. Then, the closest clusters were merged by means of a serial algorithm. Each step of the merging algorithm provided a partition of the population into homogeneous clusters (low within-variability) that were different from the others (high between-variability). These partitions are displayed graphically on a tree diagram, where the height of the branch represents the distance between the clusters; therefore, the user sees at once which partitions have a high discriminative power. If several partitions have a similar discriminative power, the final choice is led by the clinical relevance of these partitions.

Finally, all original variables were described in the resulting groups. Clinically relevant differences between groups were tested with Student’s t-test for quantitative variables and with a chi-squared test for qualitative variables.

## Results

### Summary of results

#### Population characteristics

**Fig 1:**
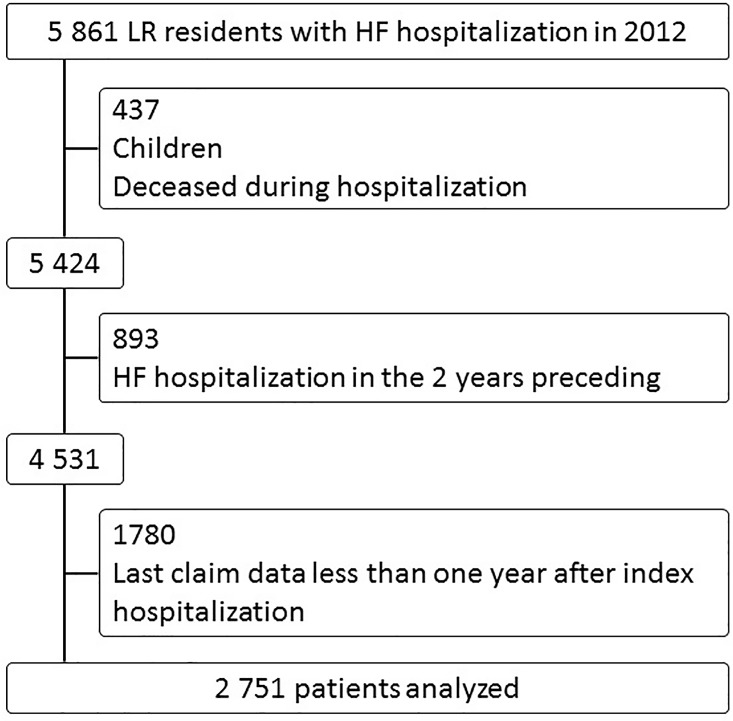
Flow Diagram.

In total, 5,851 patients residing in the LR region were admitted for HF in 2012. Among them, 437 were excluded because they were less than 18 years old or because they died during the index stay, 893 were excluded because they had an HF hospitalization during the two preceding years, and 1,780 were excluded because their follow-up was shorter than 1 year (Fig 1). Finally, 2,751 patients were included in the analysis, representing 47% of all patients hospitalized for HF in 2012 in the LR region. Population characteristics are described in Tables 1 and 2. The mean age was 78; 34% of patients had a Charlson index of 1 (i.e., only their HF), and 29% had a Charlson index of 2. During the mean follow-up time of 22 months, 18% of the patients died, and 70% were never readmitted for HF.

**Table 1:** Population characteristics (quantitative variables).

Variable	N	Mean	StD	Min	Median	Max
**Follow-up time (m)**	2,751	21.7	4.2	12	21.7	33.5
**Age (y)**	2,751	78.31	11.82	19.46	81.13	101.47
**Deprivation index**	2,541	0.4	0.63	-2.52	0.5	1.96
**Delay to 1st GP visit (d)**	2,433	18.64	36.74	0	7	559
**Delay to 1st cardiologist visit (d)**	855	84.05	124.33	0	36	744
**Mean GP delay (d)**	2,606	32.89	29.41	0	25.82	532
**Mean cardiologist delay (d)**	609	134.35	110.5	0	110.25	651
**Unforeseenness index**	2,751	6.66	9.26	0	4.35	100
**Nursing care index**	2,328	35.03	41.07	0.11	10.05	100
**Diuretic variability**	2,458	73.07	40.89	0	64.33	248.38
**Delay to treatment discontinuation (d)**	1,689	149.47	154.9	0	94	765

**Table 2:** Population characteristics (qualitative variables).

**variable**	** **	**N**	**Column %**
**Death during follow-up**		484	18
**Sex (male)**		1,375	50
CMU / AME*		130	5
**HF Readmission**	None	1,933	70
	One	524	19
	At least 2	294	11
**Charlson index**	1	937	34
	2	808	29
	3	507	18
	4	267	10
	5 or more	232	8

* Government health insurance programs for individuals with limited financial resources

#### PCA

The PCA showed that a high age, a high nursing care index and a low GP mean delay were positively linked together, and they were independent of the unforeseenness index. Delay to first GP visit was correlated with both groups of variables. Diuretic variability and delay to treatment discontinuation were independent. Detailed results are displayed in S1 File.

#### HAC

**Fig 2:**
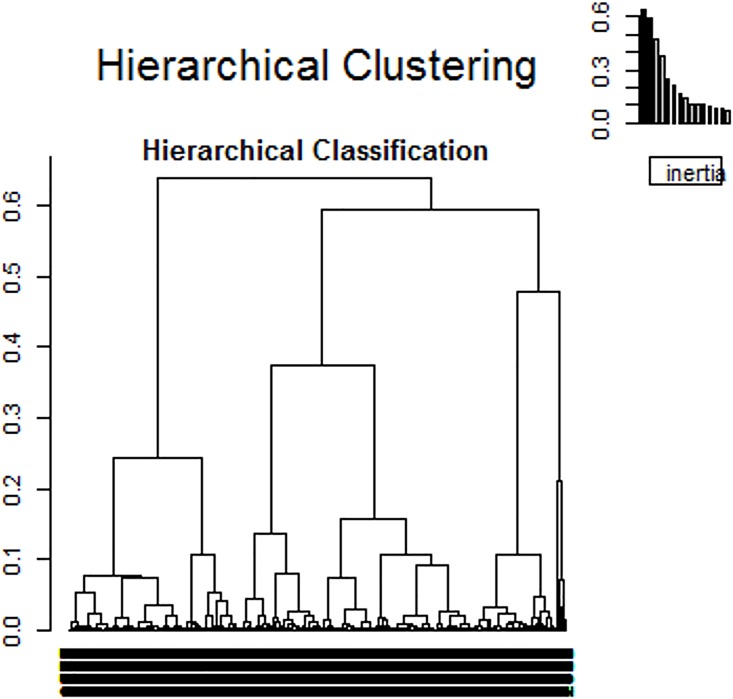
Hierarchical ascendant classification tree.

**Fig 3:**
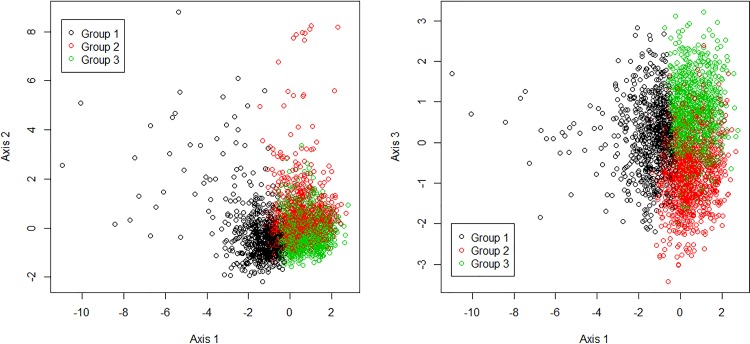
Scatter plot of individuals in the first two plans of the MCA.

The HAC was performed on the first 5 axes, which represented 70% of the variance. A 3-cluster solution provided the most descriptive power for the data (Fig 2). Variables for the groups are described in Table 3. Patients were not evenly distributed across the groups: only 27% of the total ended up in group 1, whereas more than a third (36% and 39%) were in each of groups 2 and 3, respectively. The groups are represented on the three first axes of the MCA (Fig 3). Most of the variables followed a similar pattern with relatively similar values in groups 2 and 3 and values in group 1 that were far from the means. For example, the mean age was 67 y, 82 y and 83 y in groups 1, 2, and 3, respectively. The other variables had relatively similar values in groups 1 and 3 or had different values in each group.

**Table 3:** Group characteristics.

		Group 1 N = 734	Group 2 N = 1,060	Group 3 N = 957
*Age (y)		66.84 (12.66)	81.98 (8.74)	83.05 (7.47)
*Deprivation index		0.34 (0.64)	0.38 (0.64)	0.48 (0.59)
*Delay to 1st GP visit (d)		33.4 (61.08)	14.95 (22.43)	11.91 (17.5)
**Delay to 1st cardiologist visit (d)**		89.03 (136.26)	73.86 (106.21)	89.23 (127.69)
*Mean GP delay (d)		53.52 (46)	26.29 (14.38)	24.18 (12.8)
**Mean cardiologist delay (d)**		129.41 (107.47)	142.84 (122.8)	131 (99.01)
*Unforeseenness index		5.02 (6.01)	8.16 (12.47)	6.25 (6.46)
*Nursing care index		6.56 (15.18)	35.3 (39.92)	53.94 (43.12)
*Diuretic variability		58.28 (33.74)	72.98 (42.16)	83.56 (40.96)
*Delay to treatment discontinuation (d)		144.54 (146.15)	438.77 (153.7)	117.58 (120.9)
**Death**		55 (7)	221 (21)	208 (22)
**Sex (male)**		490 (67)	494 (47)	391 (41)
**CMU / AME** ***†***		76 (10)	26 (2)	28 (3)
**HF Readmission**	**None**	612 (83)	630 (59)	691 (72)
** **	**One**	87 (12)	265 (25)	172 (18)
** **	**At least 2**	35 (5)	165 (16)	94 (10)
**Charlson index**	**1**	307 (42)	336 (32)	294 (31)
** **	**2**	208 (28)	308 (29)	292 (31)
** **	**3**	126 (17)	205 (19)	176 (18)
** **	**4**	47 (6)	110 (10)	110 (11)
** **	**5 or more**	46 (6)	101 (10)	85 (9)

Values are the mean (std) for quantitative variables or number (column percent) for qualitative variables.

* Active variables (used to perform the clustering method)

† Government health insurance programs for individuals with limited financial resources

### Interpretation of results

Group 1. Group 1 patients were mainly characterized by their age, which was, on average, 15 years younger than the two other groups (p < 10^−15^); their Charlson index was also lower (p < 10^−7^). They received less general care (long GP visit delays and low nursing care index, all p < 10^−14^) but not less cardiologist care (p ≈ 0.40) as the other groups of patients, and their diuretic delivery was less variable (p < 10^−15^). Thus, group 1 was described as “low need—low intensity of care”. Finally, they had fewer unscheduled visits (p < 10^−11^) and HF readmissions (p < 10^−35^).

Groups 2 and 3. The second and third groups of patients were comparable in terms of age and Charlson index, which were both higher than for group 1 and denoted more medical needs. Group 2 differed from group 3 by its lower quantity of general care and lower diuretic variability (p ≤ 0.001). On this basis, group 2 was described as “high need—low intensity” and group 3 as “high need—high intensity”. Interestingly, although ages or comorbidities were not significantly different between groups 2 and 3, patients in group 2 had a higher rate of unforeseen medical contacts and HF readmissions compared with group 3 (p < 0.0001).

### Validation of results

As no independent data set was available, we randomly split our data set in two groups, and replicated the HAC on each one. This analysis is reported in S2 File. It supports our main results.

## Discussion

### Findings

In this study, an exploratory approach was taken to describe the primary care management of HF patients in a French region. The description focused on three concepts: baseline clinical status, practice style, and clinical outcomes. We found significant differences in primary care management among the HF patients. Group 1 exhibited less medical need and lower intensity of care; patients in groups 2 and 3 had more medical needs but differed in terms of intensity of care and clinical outcomes. The results suggested that these three concepts mattered in describing separable and homogenous HF patient groups, and they were sufficient to determine meaningful groups. Importantly, this approach allowed discrimination of three different groups with significantly different medical needs and intensity of care, which could help practitioners manage patients better and could help the health care system in counterbalancing discrepancies.

Practice style results from numerous individual choices made by physicians, patients, and from the meeting of these choices. These choices are unobservable in claims data and may depend on preferences, opportunities and constraints [24,25]. We characterized practice style by visit delays (first post-discharge visit or mean interval with the GP or cardiologist separately), nursing care index, diuretic variability, and delay to treatment discontinuation. These variables mainly represented the intensity of practice and are practical measures used in health system monitoring [26]. The “high responsibility” and “low responsibility” practice patterns, as described by McGrail [14], were partly defined by such variables.

Low intensity can be explained by two very different causes: a low medical need or a lack of mandatory care. To address this question, these variables were interpreted in light of baseline clinical status and clinical outcomes. The size of the imbalance of clinical outcomes and of medical need in group 1, comparatively with the other two (absolute risk of death and absolute risk of HF readmission: + 15% in groups 2 and 3 versus in group 1, p < 10^−35^), suggested that medical need was the most important explanatory factor for clinical outcomes in our population. Furthermore, the difference between groups 2 and 3 (absolute risk of HF readmission: + 10% in group 2 versus group 3, p < 0.0001), in spite of similar medical need, could be attributed to the differences in practice style.

### Clinical Practice and Clinical Research implications

All our findings taken together suggested new research questions to find the optimum effect of practice intensity on clinical outcomes. In the present population with HF, the main medical cause for diuretic variability may be the treatment of acute HF. In particular, in the group 3, which receives a high intensity practice style, the high variability could denote frequent adaptations of the diuretic dose to congestive symptoms. May this explanation be confirmed by an *ad hoc* designed study, this variable could be included in measures of the reactivity of the primary care system, and help to explore the role of this reactivity in clinical outcomes.

We could not grade the severity, know the etiology, or describe the precise management of HF because the data were not available; i.e., particularly, the NYHA, the natriuretic peptides, the ejection fraction, the context of the interventional cardiological act or heart surgery, patient education and rehabilitation. These data would certainly have led to a more precise clustering. But because of the descriptive nature of our analysis, our results remain valid. This analysis would need to link clinical research data with claim data.

### Policy and Health Services Research implications

The supply in primary care is an important question in Health Services Policy. In our study, the problem is the low intensity of care. This could be linked to a low primary care supply, or to an inefficient use of a sufficient supply. A geographical refining of our results could help discriminate these two situations. This could help to choose between Health Policy fostering a better organization of care where the primary care supply is high, and development for instance of telemedicine where the primary care supply is low.

The effectiveness of primary care depends on opportunities and constraints, such as supply [12], geographical proximity [27], and financial ability [12], but also on its organization [6]. The latter is described through continuity of care [28], practice style [14] and now through practice intensity, but it probably covers far more elements. Three questions should be addressed. First, how to measure the specific performance of primary care–for example, could we measure its ability to early diagnose an acute HF? Second, which constraints, opportunities, and preferences, determine this performance? Third, which health policies could influence these elements?

### Discussion of methods

We choose to perform a multivariate descriptive analysis; this type of statistical analysis is rarely used in clinical and epidemiological research and has two major advantages. First, the crucial point is that a multivariate descriptive analysis avoids defining an a priori causal model, by contrast to a classical method, i.e. a multivariate regression model, which requires one. In our research question, it would be difficult to build a conceptual framework that is simple enough to compute a regression model: 1/ logical links between variables are complex; 2/ the concepts we aimed to study needed several variables to be described. The second advantage of our method is that it gave quantitative insight into the relevant and homogenous patients groups, which is important for a public health decisions.

We defined our variables considering the entire study period. In other words, this was a transversal study, in which we could not assume temporality between the variables. We made this choice for two reasons. First, as explained above, as we did not want to assume causal links, we did not need to control the temporality of the variables. Second, we needed a long period to define stable practice style variables. With a median study period of only 22 months, the definition of temporally ordered variables would have led us to censor a large part of our data. We thought that such a choice would greatly impair the internal validity of the study.

Patients who had been hospitalized for HF during the two preceding years or were followed-up for less than 1 year were not included. Hence, patients in the study sample might have a less severe HF than the general hospitalized HF population, and these results should not be transferred to the most severe group of patients. We chose these inclusion criteria to select patients with data available for analysis, as well as patients who were newly treated in a hospital setting for HF, to depict a global population as homogeneous as possible, and to enable us to distinguish consistent profiles. Quantitatively, our study population represented only half of all patients hospitalized for HF. This weakness is shared by recent studies assessing practice style features [29].

A more stringent problem was the patients who were never hospitalized and were therefore invisible to our data collection procedure. In the severity continuum of the disease, little is known about the ability of a hospitalization to correctly indicate a more serious status compared to the basal status. Therefore, it is difficult to evaluate the selection bias driven by this feature of our data.

Claims data contain medication dispensation; however, this is only a proxy of medication intake. In our case, the therapeutic classes of interest were not dispensed over-the-counter, so the measurement bias could have only overestimated the actual intake.

The distinction between HF with a reduced or preserved ejection fraction is an important clinical feature regarding the recommended long-term treatment, and it was not registered in our database. Indeed, the ICD-10, currently used in France, does not describe various HF features. However, the potential classification bias was evenly shared among patients and could only weaken our results. We attempted to partially overcome this problem by considering the proxy of the hospital prescription (*i*.*e*., the first prescription after discharge) as the recommended treatment.

We were interested in the discontinuation of long-term treatment because it is a known cause of cardiovascular events [30]. Unfortunately, our results were not consistent with other variables; our discontinuation delay might not have correctly measured the concept of persistence. This could be due to excessive simplification of a complex therapeutic strategy, as we aimed to include all therapeutic classes in our variables.

These inconveniences were balanced by major advantages including the ability to analyze an exhaustive population, without volunteering bias, during a long period, and with a nearly null rate of lacking data. Therefore, it is currently the best database for studying the features of primary care delivery. In the future, our results will need to be confirmed and developed by studies integrating other measures such as clinical severity, etiology, local health care supply, and preferences of actors. Such empirical studies should be conducted simultaneously with the development of a conceptual framework for the performance of primary care.

## Conclusion

This study shed light on the role of primary care practice styles in the management of patients with HF. In higher need patients, a low-intensity practice style seemed associated with poorer clinical outcomes.

Hence, pursuing the efforts of front-line specialized networks, several measures may be taken to improve outpatient care, such as financial incentives to promote an effective multidisciplinary preventive follow-up, secure and pragmatic tools to permit easy data sharing between stakeholders, and educational programs to teach health professionals how to use these opportunities.

Furthermore, we strongly advocate that additional research should be conducted on the “physiology” of primary care.

## Supporting Information

S1 File Detailed results of the PCA. (DOCX)

S2 File Validation of the HAC. (DOCX)

S3 File Pre-Processed data. (CSV)
