# Supplementary material for: The Intensity of Primary Care for Heart Failure Patients: A Determinant of Readmissions? The CarPaths Study: A French Region-Wide Analysis
Source: PLoS One. 2016 Oct 11;11(10):e0163268. doi: 10.1371/journal.pone.0163268 (PMC5058477; doi:10.1371/journal.pone.0163268)

**S1: Detailed results of the PCA**

As the PCA applies to quantitative variables, we included age, deprivation index, delay to first GP visit, GP mean delay, unforeseenness index, diuretic variability, and delay to treatment discontinuation. We did not include the delay to first cardiologist visit or the cardiologist mean delay because less than a third of the patients were referred to a cardiologist during follow-up.

The Table 1 shows the numerical results. The contributions show which variables contributed the most to each axis or dimension; the cos² shows the correlation between variables and axes; the coordinates show which variables were correlated. The correlation circles show variables as vectors, defined by the coordinates. The variables close to the circle are well represented on this plane, which allows the interpretation of their relative position. The first circle (Figure 1) shows that age and nursing care index were positively correlated and negatively correlated to GP mean delay, respectively. These variables are independent of the unforeseenness index, and the delay to first GP visit was correlated to both GP mean delay and the unforeseenness index. The second circle (Figure 2) shows that delay to treatment discontinuation was independent of diuretic variability.

The scree plot (Figure 3) shows the percentage of total variance absorbed by each axis. There was no slope failure, indicating that there was probably not much basal noise in our data. The five first axes absorbed 72% of the total variance.

Table 1: Numerical results

|  | Coordinates | | | | | Cos2 | | | | | Contribution | | | | |
| --- | --- | --- | --- | --- | --- | --- | --- | --- | --- | --- | --- | --- | --- | --- | --- |
|  | Dim 1 | Dim 2 | Dim 3 | Dim 4 | Dim 5 | Dim 1 | Dim 2 | Dim 3 | Dim 4 | Dim 5 | Dim 1 | Dim 2 | Dim 3 | Dim 4 | Dim 5 |
| Age | **0.67** | 0.26 | -0.18 | -0.04 | 0.26 | 0.45 | 0.07 | 0.03 | 0.00 | 0.07 | **29.53** | 6.03 | 3.13 | 0.14 | 7.35 |
| Deprivation index | 0.05 | 0.07 | **0.50** | **0.78** | 0.16 | 0.00 | 0.00 | 0.25 | 0.61 | 0.03 | 0.15 | 0.42 | **23.67** | **60.44** | 2.85 |
| Delay to 1st GP visit | -0.39 | **0.50** | -0.04 | -0.33 | 0.16 | 0.15 | 0.25 | 0.00 | 0.11 | 0.03 | 10.19 | **22.74** | 0.12 | 10.96 | 2.90 |
| GP mean delay | **-0.68** | 0.16 | 0.01 | -0.02 | 0.15 | 0.47 | 0.03 | 0.00 | 0.00 | 0.02 | **30.62** | 2.42 | 0.01 | 0.03 | 2.26 |
| Unforeseenness index | 0.05 | **0.78** | 0.07 | 0.12 | 0.17 | 0.00 | 0.61 | 0.00 | 0.01 | 0.03 | 0.17 | **54.69** | 0.42 | 1.44 | 3.10 |
| Nursing care index | **0.60** | -0.02 | 0.10 | -0.26 | 0.33 | 0.37 | 0.00 | 0.01 | 0.07 | 0.11 | **24.07** | 0.05 | 0.94 | 6.87 | 11.73 |
| Diuretic variability | 0.23 | 0.28 | **0.58** | -0.27 | **-0.66** | 0.05 | 0.08 | 0.33 | 0.07 | 0.43 | 3.49 | 6.81 | **31.63** | 7.15 | **46.16** |
| Discontinuation delay | 0.16 | 0.28 | **-0.65** | 0.36 | **-0.47** | 0.03 | 0.08 | 0.42 | 0.13 | 0.22 | 1.77 | 6.85 | **40.09** | 12.97 | **23.65** |

Figure 1: Correlation circle of axes 1 and 2


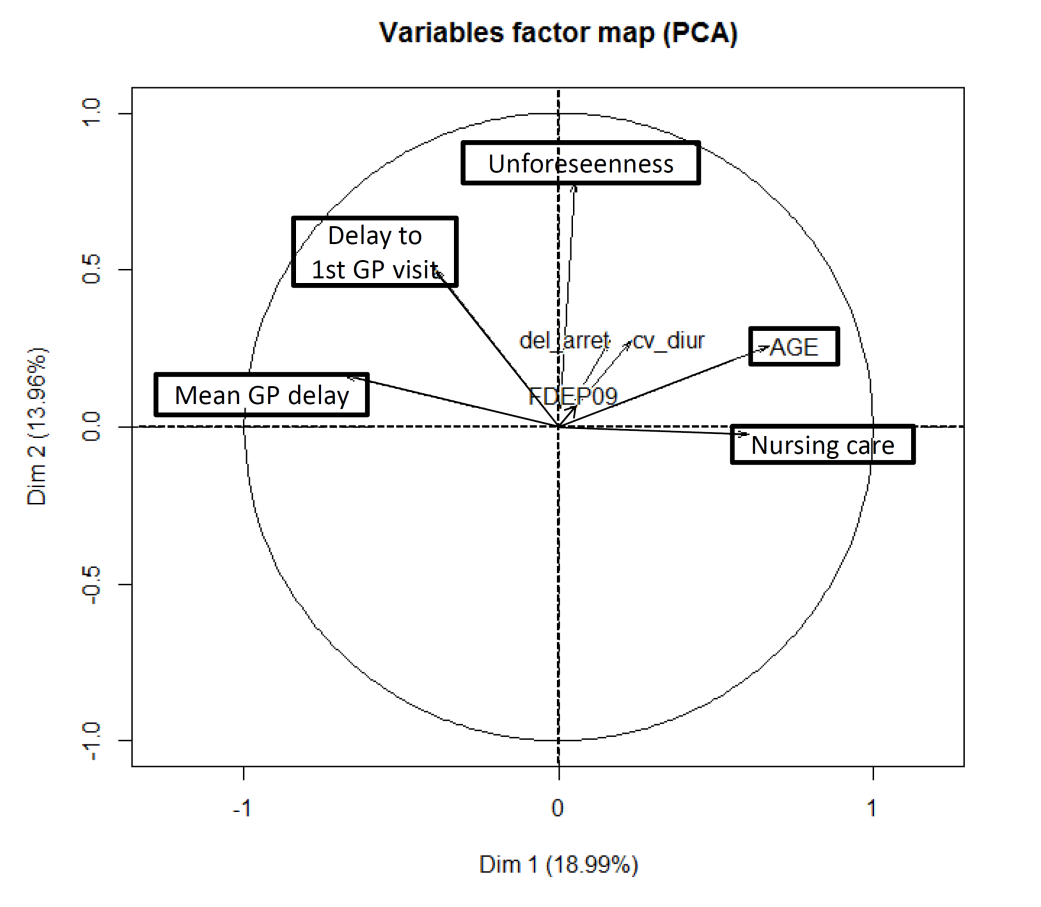


Figure 2: Correlation circle of axes 3 and 5


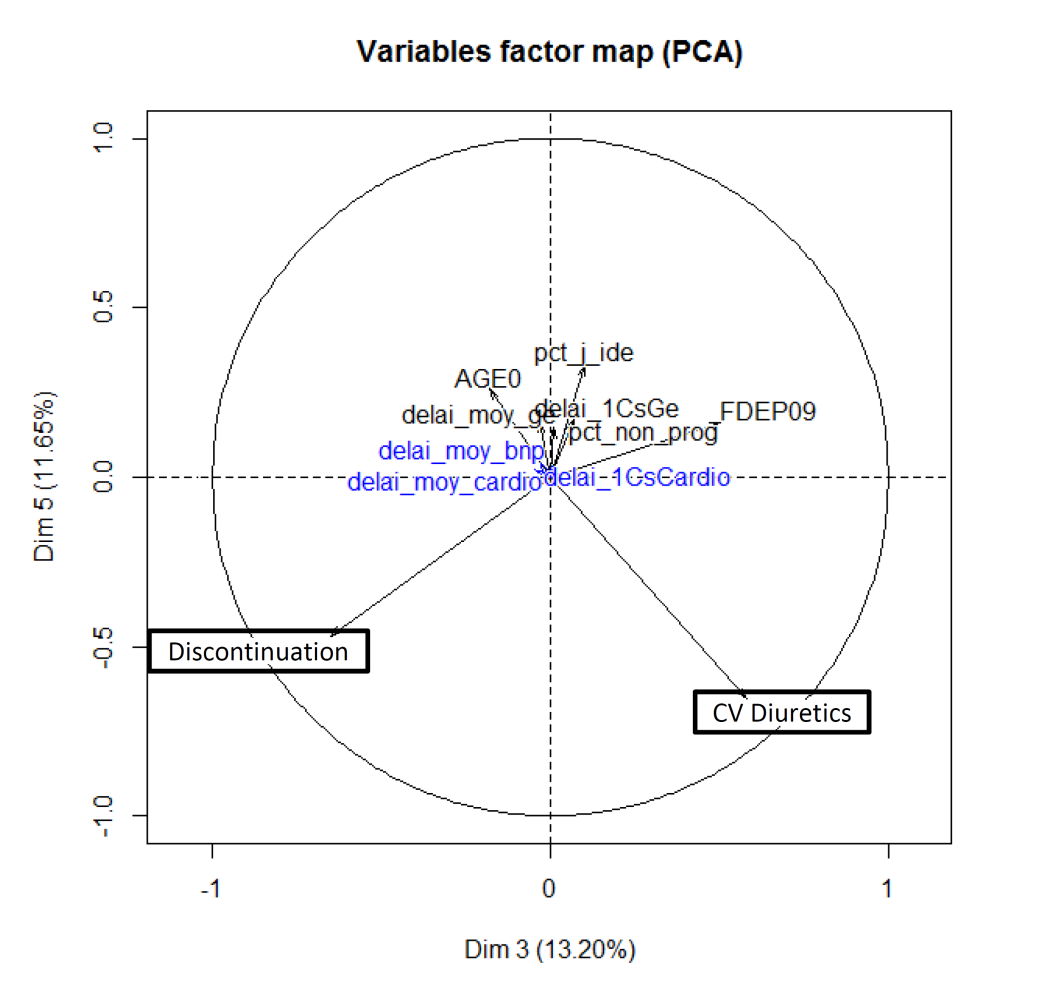


Figure 3: Scree plot


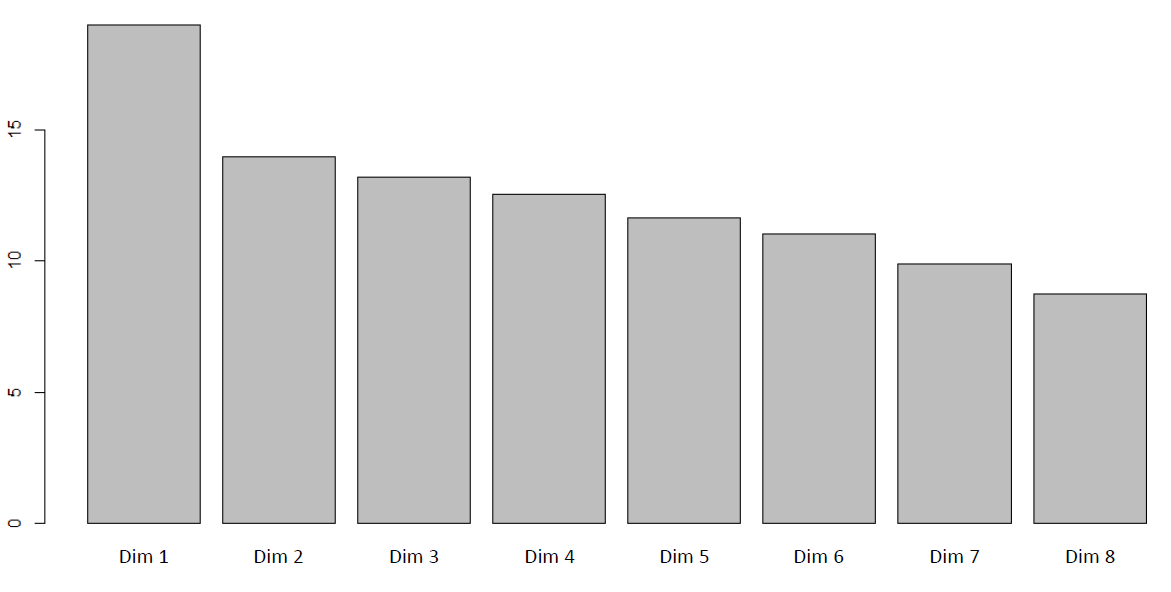

Supplement: S1 File — (DOCX) [file pone.0163268.s001.docx]
