# Supplementary material for: The Intensity of Primary Care for Heart Failure Patients: A Determinant of Readmissions? The CarPaths Study: A French Region-Wide Analysis
Source: PLoS One. 2016 Oct 11;11(10):e0163268. doi: 10.1371/journal.pone.0163268 (PMC5058477; doi:10.1371/journal.pone.0163268)
Supplement: S2 File — (DOCX) [file pone.0163268.s002.docx]

**S2: Validation of the HAC**

In order to assess the reliability of our results (original HAC), we randomly split our data set in 2 parts, and applied a HAC on each part (HAC 1, HAC 2). The objective was to retrieve a 3-groups clustering that matched the clustering of the whole data set.

Part 1 (n=1375):

The dendrogram (Figure 4) shows the early separation of a residual group, that we will not describe. As the 4-groups clustering had not a high variance gain, we choose the 5-groups clustering.

Figure 4 : Dendrogramm of HAC 1


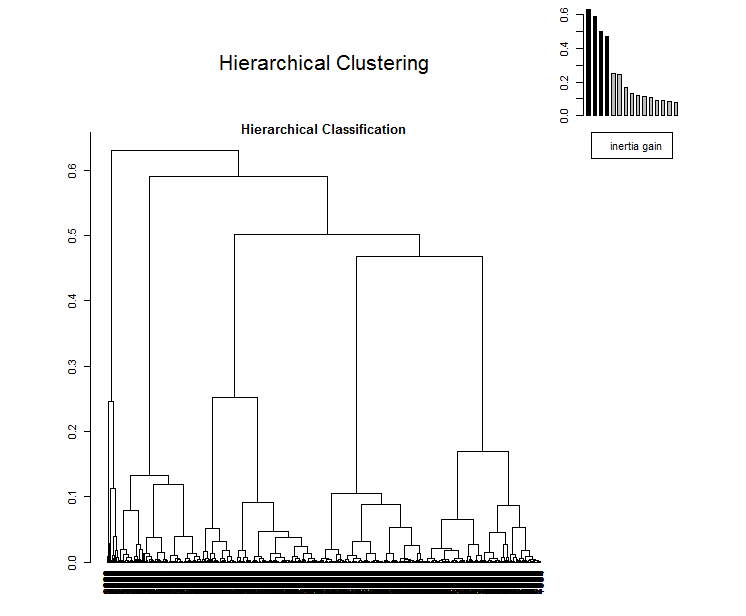


As detailed in Table 2, 77% of patients who were in Group 1 are in the Group B of the new HAC; 63% of Group 2 patients are in the Group C, and 89% of Group 3 patients are dispatched in Groups D and E.

Table 2: Contingency table of membership of patients of the Part 1, in the original HAC and in the HAC 1.

|  |  | Groups of HAC 1 | | | | |  |
| --- | --- | --- | --- | --- | --- | --- | --- |
|  |  | A | B | C | D | E | Total |
| Groups of original HAC | 1 | 31 | **286** | 24 | 20 | 12 | 373 |
|  | 2 | 20 | 0 | **329** | 94 | 80 | 523 |
|  | 3 | 1 | 53 | 0 | **107** | **318** | 479 |
|  | Total | 52 | 339 | 353 | 221 | 410 | 1375 |

The description of original variables in the groups of the HAC 1 is in Table 3. The patients of Group B are younger and have less comorbidities. Their intensity of care (delays, nursing care index, diuretic variability) is lower, and they have less clinical outcomes (unforeseen care, hospitalization, death). Now, the differences between the Group C and the Groups D+E include a younger age and slightly less comorbidities in the Group C. The Group C has a lower intensity of care. However, the differences in clinical outcomes are far less important than in the original HAC.

Table 3: description of the groups of HAC 1

|  | Group A  (n = 52) | | Group B  (n = 339) | | Group C  (n = 353) | | Group D  (n = 221) | | Group E  (n = 410) | | Groups D+E |
| --- | --- | --- | --- | --- | --- | --- | --- | --- | --- | --- | --- |
|  | Mean  or n | SD  or % | Mean  or n | SD  or % | Mean  or n | SD  or % | Mean  or n | SD  or % | Mean  or n | SD  or % | Weighted mean or % |
| Age (y) | 70,22 | 18,8 | **68,06** | 12,74 | **79,75** | 10,01 | 79,12 | 10,17 | 85,24 | 6,25 | **83,1** |
| Deprivation index | 0,43 | 0,63 | **0,51** | 0,59 | **0,36** | 0,66 | 0,44 | 0,63 | 0,34 | 0,62 | **0,38** |
| Delay to 1st GP visit (d) | 107,32 | 94,13 | **19,7** | 25,47 | **13,85** | 19,08 | 16,27 | 21,67 | 13,06 | 18,82 | **14,18** |
| Delay to 1st cardiologist visit (d) | 66,43 | 79,88 | **90,57** | 141,98 | **83,8** | 132,82 | 63,6 | 96,66 | 87,65 | 121,36 | **79,23** |
| Mean GP delay (d) | 148,21 | 135,6 | **44,93** | 25,28 | **27,92** | 15,03 | 24,54 | 13,23 | 24,19 | 12,99 | **24,31** |
| Mean cardiologist delay (d) | 131,62 | 79,36 | **136,97** | 106,48 | **147,22** | 135,03 | 118,98 | 104,51 | 124,84 | 93,66 | **122,79** |
| Unforeseenness index | 29,45 | 28,34 | **4,32** | 5,1 | **6,27** | 7,21 | 6,75 | 5,9 | 5,99 | 5,5 | **6,26** |
| Nursing care index | 4,37 | 12,72 | **6,24** | 16,76 | **12,76** | 21,39 | 32,71 | 38,19 | 65,32 | 42,07 | **53,9** |
| Diuretic variability | 70,53 | 43,88 | **55,95** | 25,82 | **56,01** | 26,97 | 139,97 | 26,81 | 63,29 | 26,57 | **90,15** |
| Delay to treatment discontinuation (d) | 379,15 | 336,27 | **171,85** | 206,99 | **737,34** | 90,23 | 395,25 | 322,04 | 244,46 | 270,08 | **297,27** |
| Death | 12 | 23 | 270 | **8** | 64 | **18** | 46 | 21 | 85 | 21 | **21** |
| ≥ 1 HF Readmission | 15 | 29 | 46 | **13** | 126 | **36** | 106 | 50 | 111 | 27 | **34** |
| Charlson index |  |  |  |  |  |  |  |  |  |  |  |
| 1 | 15 | 29 | 145 | **43** | 117 | **33** | 63 | 29 | 120 | 29 | **29** |
| 2 | 19 | 37 | 90 | **27** | 113 | **32** | 60 | 27 | 136 | 33 | **31** |
| 3 | 8 | 15 | 58 | **17** | 68 | **19** | 47 | 21 | 80 | 20 | **20** |
| 4 | 7 | 13 | 19 | **6** | 31 | **9** | 26 | 12 | 41 | 10 | **11** |
| 5 | 3 | 6 | 27 | **8** | 24 | **7** | 25 | 11 | 33 | 8 | **9** |

Part 2 (n=1376):

The dendrogram (Figure 5) shows the early separation of a residual group, that we will not describe. We choose the 4-groups clustering.

*Figure 5 : Dendrogramm of HAC 2*


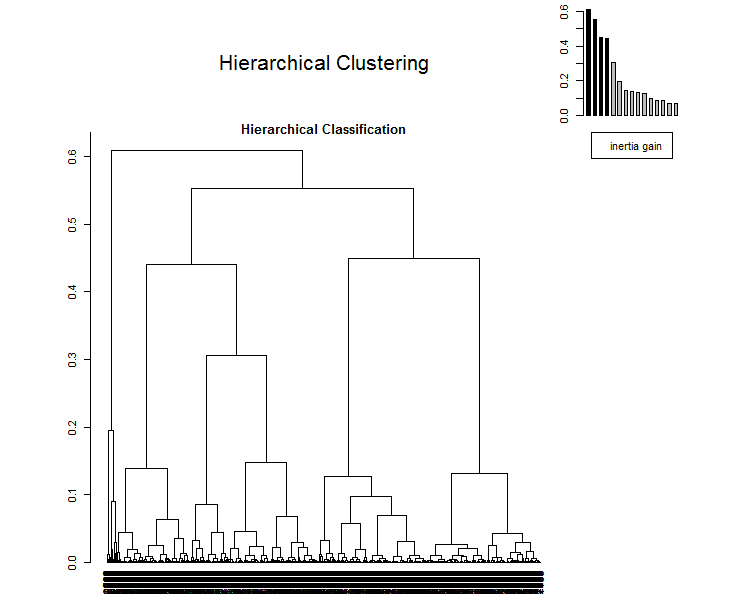


As detailed in Table 4, 92% of patients who were in Group 1 are in the Group B of the new HAC; 92% of Group 2 patients are in the Group C, and 84% of Group 3 patients are dispatched in Groups D and E.

*Table 4: Contingency table of membership of patients of the Part 2, in the original HAC and in the HAC 2*

|  |  | Groups of HAC 2 | | | |  |
| --- | --- | --- | --- | --- | --- | --- |
|  |  | A | B | C | D | Total |
| Groups of original HAC | 1 | 16 | **331** | 12 | 2 | 361 |
|  | 2 | 11 | 13 | **494** | 19 | 537 |
|  | 3 | 0 | 71 | 5 | **402** | 478 |
|  | Total | 27 | 415 | 511 | 423 | 1376 |

The description of original variables in the groups of the HAC 2 is in Table 5. The patients of Group B are younger and have less comorbidities. Their intensity of care (delays, nursing care index, diuretic variability) is lower, and they have less clinical outcomes (unforeseen care, hospitalization, death). The differences between the Group C and the Groups D+E still include a younger age and slightly less comorbidities ind the Group C, though less important than in HAC 1. The Group C has a lower intensity of care. However, the differences in clinical outcomes are less important than in the original HAC : only the rehospitalization rate is higher in Group C, and the unforeseenness index is even slightly lower in Group C.

Table 5 : Description of the groups of HAC 2

|  | Group A (n = 27) | | Group B (n = 415) | | Group C (n = 511) | | Group D (n = 423) | |
| --- | --- | --- | --- | --- | --- | --- | --- | --- |
|  | Mean or n | SD or % | Mean or n | SD or % | Mean or n | SD or % | Mean or n | SD or % |
| Age (y) | 81.22 | 11.80 | **68.75** | 11.86 | **82.23** | 8.09 | **83.65** | 7.58 |
| Deprivation index | 0.36 | 0.61 | **0.43** | 0.61 | **0.31** | 0.66 | **0.46** | 0.60 |
| Delay to 1st GP visit (d) | 252.47 | 155.71 | **18.09** | 24.30 | **14.05** | 18.80 | **12.60** | 19.07 |
| Delay to 1st cardiologist visit (d) | 170.00 | 175.03 | **86.57** | 130.44 | **78.58** | 119.70 | **81.85** | 104.42 |
| Mean GP delay (d) | 99.04 | 66.94 | **43.98** | 29.57 | **27.44** | 15.31 | **24.13** | 13.55 |
| Mean cardiologist delay (d) | 198.92 | 87.77 | **122.28** | 107.21 | **142.13** | 113.02 | **142.68** | 111.84 |
| Unforeseenness index | 36.32 | 36.90 | **4.23** | 4.37 | **6.66** | 7.15 | **7.14** | 7.34 |
| Nursing care index | 16.09 | 36.73 | **6.57** | 14.86 | **26.74** | 37.66 | **56.44** | 44.31 |
| Diuretic variability | 61.83 | 34.38 | **57.58** | 32.24 | **68.13** | 37.96 | **91.09** | 42.84 |
| Delay to treatment discontinuation (d) | 398.33 | 316.11 | **216.86** | 238.54 | **733.05** | 108.97 | **150.64** | 190.71 |
| Death | 4 | 15 | 39 | **9** | 111 | **22** | 96 | **23** |
| ≥ 1 HF Readmission | 4 | 15 | 83 | **20** | 196 | **38** | 131 | **31** |
| Charlson index |  |  |  |  |  |  |  |  |
| 1 | 8 | 30 | 173 | **42** | 170 | **33** | 126 | **30** |
| 2 | 8 | 30 | 119 | **29** | 149 | **29** | 114 | **27** |
| 3 | 7 | 26 | 60 | **14** | 95 | **19** | 84 | **20** |
| 4 | 2 | 7 | 35 | **8** | 52 | **10** | 54 | **13** |
| 5 | 2 | 7 | 28 | **7** | 45 | **9** | 45 | **11** |

Conclusion

These two HAC validate mostly the results of the original HAC. The Group 1 keeps its features. The differences and similarities between the Groups 2 and 3 are less distinctive, but remain grossly the same. This validation method confirms the reliability of the original analysis.
